# Reproductive labor, social vulnerability, and female aging: a scoping review

**DOI:** 10.3389/fsoc.2026.1767534

**Published:** 2026-07-20

**Authors:** Paulo Henrique Souza Roberto, Isabelle Patriciá Freitas Soares Chariglione

**Affiliations:** 1 Laboratory of New Epistemologies and Human Development, Institute of Psychology, Postgraduate Program in Developmental and School Psychology (PGPDE), University of Brasília, Brasília, Brazil; 2 Laboratory of New Epistemologies and Human Development, Institute of Psychology, Postgraduate Programs in Developmental and School Psychology (PGPDE) and Social, Work and Organizational Psychology (PSTO), University of Brasília, Brasília, Brazil

**Keywords:** aging in women, domestic work, homemaker, reproductive labor, social vulnerability

## Abstract

**Introduction:**

Reproductive labor, essential to social and biological maintenance, has historically been performed by women, often under conditions of social vulnerability and without formal recognition. This scoping review aimed to map the international scientific literature on paid and unpaid domestic work performed by adult and older women in contexts marked by devaluation and cumulative chronic stress, manifested in health disparities.

**Methods:**

The review followed PRISMA-ScR and JBI guidelines and included articles indexed in PubMed, PsycInfo, Scopus, and Web of Science, with no time restrictions. After screening 2,122 records, 41 studies were included.

**Results:**

The findings reveal that domestic work, particularly for migrant women, serves as a critical space in which gender, class, and racial inequalities are reproduced. Most of the reviewed studies were carried out in China, Singapore, and the United States and were based mainly on qualitative or mixed-methods designs. Major themes included migration and the physical and mental health impacts of domestic work. The literature shows high prevalence of psychological distress, musculoskeletal pain, fatigue, and symbolic and physical violence, all worsened by informality and lack of legal protection. Workers' bodies and subjectivities emerge as terrains where structural inequalities are inscribed, making visible the “colonization of subjectivity” as an effect of precarization.

**Conclusion:**

Domestic work must be understood not only as an economic activity but also as a social, relational, and neurobiological phenomenon. These factors underscore the consequences of such working conditions for the health of marginalized populations. Moreover, the mapping highlights the urgent need for public policies that integrate social protection, economic recognition, and health care for these workers.

## 1 Introduction

Reproductive labor, essential to both social and biological maintenance, has historically been performed by women, often under conditions of social vulnerability and without formal recognition (Gopal and Lupo, 2025; Queji, 2024). This context of devaluation and overload imposes a cumulative burden of chronic stress that transcends the social sphere and manifests in health disparities. As women engaged in reproductive labor (such as domestic workers and homemakers) age, the intersection of the physical and emotional demands of unpaid labor and chronic social vulnerability may accelerate the senescence process (Jose et al., 2025; Krishna, 2024).

Domestic work has historically been conceptualized as reproductive labor, relegated to a position of social and economic invisibility (Federici, 2017; Rezende et al., 2025; Souza and Ferraz, 2023). The division between public and private spheres reinforced the sexual division of labor, assigning women the tasks of care and social reproduction, not being recognized as workers who generate market value (Davis, 2016; Federici, 2019; Saffioti, 1984).

The invisible and immeasurable nature of these tasks (Carrasco, 2006; Federici, 2019) shapes the lived reality of women who dedicate their entire lives to caring for others—children, family members, and even unrelated individuals. This context is marked by working conditions that generate physical, emotional, and psychological suffering due to the affective demands inherent to such labor. Thus, reproductive labor becomes psychically inscribed as an ambivalent position—one that simultaneously carries love and indifference (Coutinho et al., 2018; Mello, 2021).

For homemakers, unpaid domestic work is framed as “natural” and “indissociable” from motherhood, therefore perpetuated as part of female socialization from childhood (Federici, 2019; Torres, 1989). Although this work involves a complex set of tasks—from household hygiene to the care of others (Andrews et al., 2023; Garcia and Marcondes, 2022; Kaplan, 2022; Monteiro et al., 2018)—it remains invisible and undervalued.

The invisibility of unpaid domestic work reflects the historical marginalization of women in the public sphere, with cumulative effects across the life course, particularly in older age, when many continue to perform care roles, now also as grandmothers or “matriarchs” (Almeida, 2018; Silva, 2012).

In contrast, paid domestic work, although performed outside the home and recognized as a market activity, also reproduces inequalities of gender, class, and race. Even when wages are provided, this labor is historically precarious, viewed as a “female activity,” and characterized by informality, excessive physical demands, environments marked by violence, low wages, and lack of legal and social recognition, all of which harm the physical and mental health of these women (Aynalem et al., 2025; Azanaw et al., 2019; Beja, 2014; Ejigu et al., 2020; Gebremariam et al., 2024; Zhou, 2024).

This workforce, composed predominantly of Black women and women with low educational attainment, is aging with limited prospects for social mobility (Girard-Nunes and Silva, 2013). According to the International Labor Organization's report *Making Decent Work a Reality for Domestic Workers* (International Labour Organization, 2022), there are 75.6 million domestic workers worldwide (4.5% of all paid workers). However, 36% remain entirely excluded from labor laws and protections, underscoring the urgent need for coordinated legal frameworks, particularly in Asia, the Pacific, and the Arab States, where disparities are most severe.

Moreover, in general, a large share of domestic workers is concentrated in two regions of the world: approximately half (38.3 million) can be found in Asia and the Pacific—largely in China— while another portion (17.6 million) is located in the Americas (International Labour Organization, 2022). It is important to emphasize that this type of work is inherently asymmetrical, shaped by and within the sexual division of labor; however, this dynamic becomes even more complex when placed in context. In the case of domestic work in countries across Asia and the Pacific, it is essential to consider the condition of migration, which further exposes these workers to the contradictions of the labor sphere (Ghailani et al., 2024).

Even in countries with specific labor and social security legislation for domestic work, insufficient oversight continues to undermine protections for these workers, who often struggle to secure access to their legal rights. In this regard, the Court of Justice of the European Union ruled that employers must implement systems to record daily working hours to ensure compliance with Directive 2003/88 on working time. This decision aims to guarantee reliable monitoring of actual working hours and prevent unjust treatment of domestic workers (European Parliament Judgment of the Court (Seventh Chamber), 2018).

There are also multiple conflictual realities concerning domestic work worldwide. Africa, for example, is home to approximately 9.6 million domestic workers. This labor is vital to the livelihood of numerous families, as an estimated 15.8% of all paid female workers in Africa are employed as domestic workers (International Labour Organization, 2022). However, despite its essential nature, this work is characterized by systemic devaluation. Many women are employed informally, without access to fundamental labor rights such as minimum wage protections, medical assistance, or unemployment benefits. In some African countries, domestic workers are explicitly excluded from labor protections, whereas in others, enforcement mechanisms are weak or non-existent. Without maternity leave, healthcare, or safe working conditions, domestic workers are expected to care for others while their own basic needs remain unmet (Griffin, 2011; Phillips, 2011; Shamase and Sekaja, 2025; Vanyoro, 2019).

The reality in the Americas is similar. In the United States, the National Domestic Workers Alliance estimates that 2.2 million nannies, house cleaners, homemakers, and caregivers perform essential care labor in the country. The organization highlights that this labor is structured by class, race, and gender inequalities. As a historically feminized and subordinate occupation, domestic work in the U.S. is often performed by immigrant women from Central and South America, African American women, and economically marginalized women (National Domestic Workers Alliance, 2025).

In Latin America, an estimated 15 million individuals are employed in paid domestic work, 93% of whom are women. Domestic work accounts for 12% of women's employment in the region, and in countries such as Paraguay and Argentina, this percentage exceeds 16%. Women working in domestic work typically earn salaries equal to or less than half the average income of the general workforce. Moreover, almost 80% of domestic workers are employed informally. Informality rates are even higher in Central America and the Caribbean, exceeding 90% (International Labour Organization, 2024).

In Brazil, there are an estimated 6 million domestic workers. However, according to the Ministério do Trabalho e Emprego (2025), there was an 18.1% reduction in formal employment contracts between 2015 and 2024. The demographic profile of workers has also shifted: 47% are aged 50 years or older, and 12% are already older adults. Regarding education, most formally hired workers have completed or partially completed high school and earn, on average, one minimum wage (Ministério do Trabalho e Emprego, 2025). Although Brazil has specific legislation for domestic workers, including the Domestic Workers' Constitutional Amendment (PEC 72/2013), the lack of oversight continues to contribute to precarious working conditions in this sector. Although Brazil has specific legislation for domestic workers, including what is known in the country as the *PEC das Domésticas* (PEC 72/2013, the Domestic Workers' Constitutional Amendment), the persistent lack of oversight continues to contribute to precarious working conditions.

In Argentina, Law No. 26.844/2013 regulates domestic work and expands labor protections related to working hours, maternity leave, annual vacation, and year-end bonuses. It is estimated that approximately 1.5 million domestic workers are employed in the country, 99.3% of whom are women. Still, the informality rate remains high at 76.8%. The *Instituto Nacional de Estad*í*sticas* (INE, Chilean National Institute of Statistics) estimates that 223,000 individuals are employed as domestic workers, 55% of whom work informally. The highest concentration is in the Metropolitan Region of Santiago, reflecting increased demand in the capital, followed by Valparaíso and Biobío. Most domestic workers in Chile are between 40 and 59 years old (*Ministerio del Trabajo y Previsión Social de Chile, 2024*).

A critical gap in the literature concerns the limited number of studies addressing the neurobiological and psychophysiological mechanisms underlying the relationship between precarious reproductive labor and illness among marginalized populations. Chronic exposure to stressors associated with unrecognized reproductive labor and social marginalization elevates allostatic load, accelerating physiological wear-and-tear and contributing to significant health disparities, including cognitive and immunological outcomes (Assari et al., 2025). In-depth scientific investigation of these mechanisms is essential to move beyond descriptive accounts, providing the empirical basis necessary for preventive interventions and public policies aimed at mitigating the biological impacts of social oppression and promoting visibility and health equity for these populations.

Domestic work sustains a logic of exploitation that naturalizes women's subordination. This phenomenon is shaped by intersecting oppressions, like gender, class, race, and age, resulting in life trajectories marked by exhaustion, subordination, precarization, and illness. Therefore, examining these elements elucidates the importance of mapping and understanding the implications of this historically undervalued labor. In this context, the objective of the present study is to map international research on paid and unpaid domestic work performed by adult and older women who face social devaluation and cumulative chronic stress, which manifest in health disparities.

## 2 Materials and methods

This scoping review was conducted in accordance with the Preferred Reporting Items for Systematic Reviews and Meta-Analyses guidelines, specifically the extension for scoping reviews (PRISMA-ScR; Tricco et al., 2018), to ensure comprehensive and transparent reporting of existing gaps in the literature on domestic work. The review followed the Joanna Briggs Institute (JBI) methodology, including blinded peer assessment during the assessment of studies throughout the entire process. To enhance its rigor and validity, experts in the fields of older adults cognition and cultural differences were consulted during the development of the research protocol and the discussion of the findings. The reviewers also discussed at length the search strategy and the synthesis methods adopted. Consistent with the JBI guidance for scoping reviews (Peters et al., 2021), a formal critical appraisal of methodological quality or risk of bias of the included studies was not performed. Because this research did not involve the collection of primary data from human participants, ethics committee approval was not required.

### 2.1 Inclusion criteria

#### 2.1.1 Participants

The studies reviewed encompassed a wide diversity of participants engaged in paid and unpaid domestic work, reflecting different cultural, occupational, and family contexts. Included populations consisted of domestic workers, caregivers of older adults, and homemakers (ages 18–84) performing caregiving or household labor in settings marked by social, cultural, and economic inequalities. The presence of multigenerational experiences, from younger women seeking employment to workers transitioning toward retirement, underscored the heterogeneity of the populations represented. Studies were excluded if they: (a) did not adequately identify participants; (b) examined child labor; (c) involved household labor by women who were not homemakers; (d) were conducted with hotel maids or nurses; or (e) focused on older adults' perceptions of care.

#### 2.1.2 Concept

Paid and unpaid domestic work, categorized as reproductive labor in class-based societies, emerged across studies as a central activity predominantly carried out by women. The literature indicates that paid domestic work includes caregiving, cleaning, and support for older adults and children, performed largely by migrant women working under precarious conditions and lacking social protection (Anderson, 2000; Federici, 2019; Parreñas, 2001).

In contrast, unpaid domestic work performed by homemakers involves maintaining the household and providing family care without economic recognition, despite being essential to social reproduction. The studies analyzed highlight that such labor continues to be shaped by classism and heteronormativity, which confine some women to the private sphere of the home (Bengoa, 2016; Biroli, 2018; Hirata and Kergoat, 2007).

Both forms of domestic work are upheld by the logic of the sexual division of labor, which historically assigns caregiving, reproductive, and domestic roles to women while men remain associated with the public and productive sphere. This division is not merely functional but hierarchical, as it attributes lower social and economic value to domestic work, thereby reinforcing gender inequality (Hirata, 2016; Kergoat, 2010).

#### 2.1.3 Context

This scoping review included international scientific literature on the reproductive labor performed by adult and older women working as domestic workers and homemakers.

#### 2.1.4 Types of studies

This review included observational studies, clinical or experimental trials, and case studies focusing on the labor of domestic workers and homemakers, including specific subgroups such as migrant domestic workers, day laborers, informal or formal domestic workers, older domestic workers, workers nearing retirement, caregivers of older adults, immigrant caregivers of older adults, and homemakers.

Studies were excluded if they: (a) did not meet the established selection criteria; (b) were published in languages other than English; or (c) were published only as conference abstracts, study protocols, editorials, theses, dissertations, commentaries, or reports. Non–peer-reviewed articles and gray literature were not included. No limits were placed on publication date, allowing the identification of early studies and publication trends across decades.

### 2.2 Search strategy

As recommended for all JBI review types (Aromataris et al., 2024), a three-step search strategy was employed. An initial, limited search was conducted in PubMed, followed by an analysis of the titles, abstracts, and indexing terms of the retrieved articles. This preliminary step helped identify key words and descriptors to inform a more comprehensive search across all relevant databases.

The second search, conducted between March and June 2025, incorporated all identified search terms and descriptors. Only studies published in English were included. The databases searched were PubMed, PsycInfo, Scopus, and Web of Science. To ensure reproducibility, the exact same Boolean search string was applied to all databases without modification:

In the third step, the review was registered on the Open Science Framework (10.17605/OSF.IO/J87NR). The search strategy was adapted to the specific indexing systems of each database, using the following terms: (“Adult” OR “Middle Adulthood” OR “aged” OR “old age” OR “older adulthood”) AND (“Household Management” OR “House Cleaner” OR “Maid” OR “Domestic Service Personnel”).

Regarding the scope of the search, regional databases (e.g., LILACS, SciELO, AJOL) were not included. The decision to focus on these four major indexing services was made to capture high-impact international literature and manage the feasibility of the review given the vast volume of global records. We acknowledge that this, combined with the exclusion of non-English language studies, may limit the retrieval of locally published research from the Global South. Gray literature (e.g., conference abstracts, theses, unpublished reports) was also excluded. This decision was taken to ensure that the mapping was based on peer-reviewed studies, thereby maintaining a standard of methodological reliability and scientific validation in the synthesized evidence.

### 2.3 Study selection

Zotero was used to collect and organize studies from all databases, remove duplicates, extract relevant data, and document screening decisions. A Microsoft Excel spreadsheet was used for data extraction and analysis by two independent reviewers (PHSR and IPFSC). These reviewers screened titles and abstracts according to inclusion and exclusion criteria. Articles that met the criteria were retrieved and assessed in full by both reviewers. The selection process demonstrated high reliability, with an inter-rater agreement rate of 91.13% across the screening phases. In cases of disagreement, a third reviewer (PTS) was consulted to reach consensus.

### 2.4 Data extraction

Data were extracted using Microsoft Excel and included information about study title and authors, country and city of origin, demographic characteristics of the study population, study design, and key findings relevant to the review objective.

### 2.5 Data analysis and presentation

The selected literature was mapped in terms of quantity, type, characteristics, and sources of evidence according to the objective of this scoping review. Following the JBI Manual for Evidence Synthesis (Peters et al., 2021), the mapping process involved extracting data from the abstracts of each article (*n* = 2,122), including author, year, title, country, population characteristics, study design, and summary of main findings. Two authors (PHSR and IPFSC) extracted and mapped the data into the extraction table, and a third author (PTS) verified the extracted information.

## 3 Results

The initial search across all databases was conducted in May 2025, followed by the analysis of selected articles in June 2025. The search strategy was designed to be comprehensive and sensitive, resulting in the retrieval of a substantial number of studies. A total of 2,122 records were identified, of which 386 were excluded as duplicates, leaving 1,735 studies for title and abstract screening.

**Figure 1:**
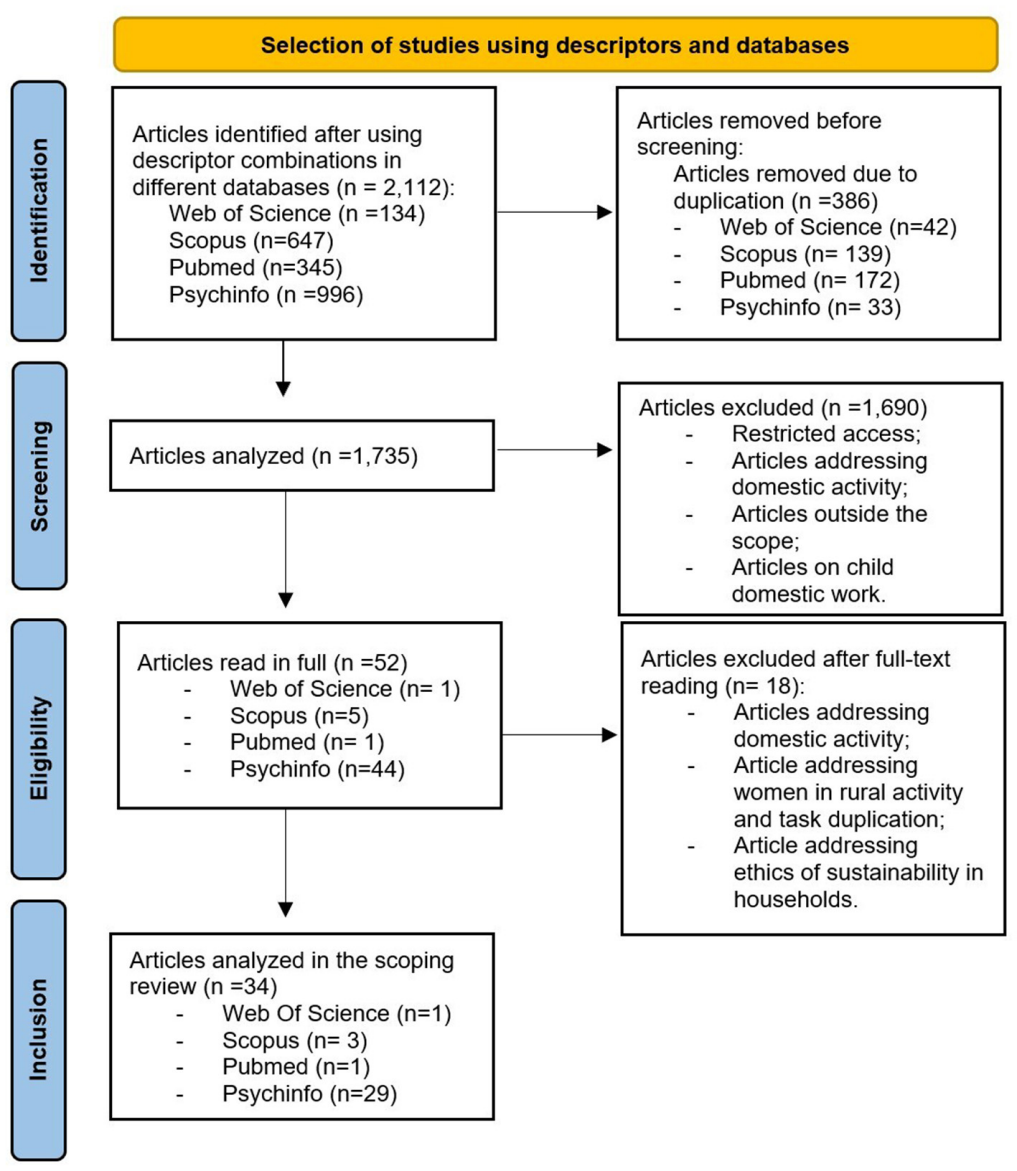
PRISMA-ScR flowchart illustrating the study selection process using descriptors and databases across the Identification, Screening, Eligibility, and Inclusion phases.

After the initial screening phase, 1,690 studies were excluded for not meeting the inclusion criteria. The 52 remaining records were assessed for full-text availability, and 18 were excluded for not meeting eligibility criteria. Thirty-four studies were ultimately included for full-text review. The PRISMA-ScR flowchart (Tricco et al., 2018), presented in Figure 1, illustrates the study selection process.

**Figure 2:**
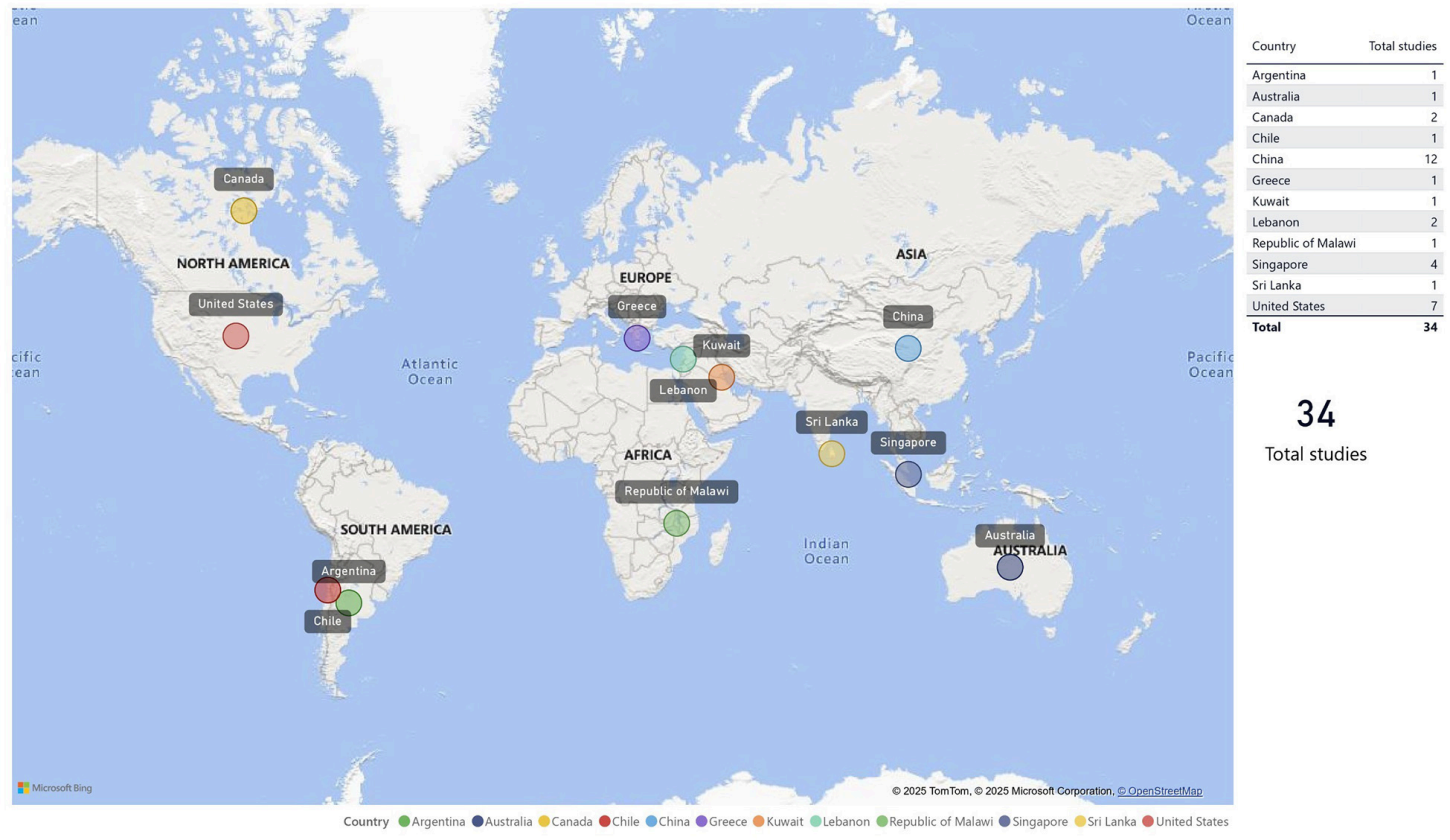
World map visualization highlighting the geographical distribution of the 34 included studies by country.

Supplementary Table 1 provides an overview of the characteristics of the reviewed studies and the main findings from this scoping review. Figure 2 displays the number of studies in which a specific cultural context was mentioned.

Regarding publication year, the temporal span ranges from 1959 to 2025, with a higher concentration of studies beginning in the 1990's and a marked increase after 2010 (*n* = 24). Recent publications reflect a research agenda aligned with contemporary discussions on migration, gender, aging, and mental health, particularly within the transnational domestic work landscape.

This upward trend is closely related to the growing academic interest in migrant domestic workers' rights, population aging, and the expanding role of domestic workers in long-term care, especially in countries and territories characterized by high immigration rates, such as Hong Kong and Singapore. This temporal pattern suggests that the field has evolved in response to global social changes, including the expansion of care economies and the influence of migration policies. It also signals methodological advancement, with increasing use of longitudinal designs, social network analyses, and mixed-methods approaches integrating quantitative and qualitative data.

Overall, the reviewed literature presents a broad and multifaceted panorama of domestic work and its social, psychological, and health-related implications across geographic and historical contexts. The analysis of publication countries reveals a concentration of studies in China (*n* = 12) and the United States (*n* = 7), followed by Lebanon (*n* = 2), Singapore (*n* = 4), and Canada (*n* = 2), with single studies from Greece, Argentina, Chile, Sri Lanka, Australia, Kuwait, and Malawi, as shown in Figure 2.

As noted earlier, 12 studies were conducted in China (particularly in Hong Kong and Macau), emphasizing the centrality of these regions in research on migrant domestic workers, especially Filipina workers, who frequently appear in conditions of heightened vulnerability and social risk. The seven studies conducted in the United States primarily examine domestic work through the lens of gender and race, highlighting structural markers shaping this occupational sphere. These studies also show greater attention to family dynamics, gender roles, and intersections with race and ethnicity, as well as how these factors influence the distribution of domestic and caregiving labor in multicultural settings. Another notable feature is the inclusion of research on aging domestic workers and their employers, as well as the effects of care work on children, older adults, and the workers themselves, contributing to an intergenerational understanding of caregiving labor.

The diversity of countries represented expands the understanding of how cultural, economic, and political factors shape domestic work in different contexts, enabling interregional comparisons and revealing shared patterns such as vulnerability, precariousness, and impacts on physical and mental health.

Methodologically, the reviewed studies employ a wide range of approaches, with qualitative and mixed-methods designs predominating. Common methods include in-depth interviews, focus groups, and ethnography. Quantitative and cross-sectional studies were also identified, using questionnaires and statistical analyses to examine associations among work demands, loneliness, mental health, and health behaviors.

The analysis of study participants is equally revealing. The studies encompass large population-based samples—such as a survey of 5,998 individuals in Lebanon and samples of over 1,000 participants in Macau—as well as ethnographic studies involving fewer than 10 women, allowing for in-depth exploration of subjective experiences. Overall, participants were predominantly women, often migrants, of working age, and living in conditions of social, economic, and/or health vulnerability. Many of these women were working in migration contexts (e.g., China, Singapore, the Philippines, Sri Lanka, and Greece) and reported mental health burdens, occupational stress, and diminished social support networks, underscoring emotional overload and coping challenges.

In essence, the reviewed studies depict a dynamic field that integrates quantitative analyses—such as the prevalence of psychiatric symptoms, overweight rates, and standardized scale assessments—with qualitative investigations of narratives, meanings, and subjective experiences. The relationship among publication countries, years, and participant profiles suggests a shift toward more complex, intersectional, and transnational analyses of domestic work, recognizing it as a phenomenon shaped by gender, migration, social class, and public policies.

The themes highlighted in these studies primarily relate to migration contexts, working conditions, and health outcomes among domestic workers. Commonly identified issues include experiences of violence, subordination, sexual harassment, and discrimination, as well as mental distress and illness resulting from the historical precarization of their working and living conditions. For homemakers, particularly older women, the findings emphasize the physical and emotional consequences of a lifetime dedicated to caregiving. These issues are further elaborated in the Discussion section.

This literature demonstrates that domestic work is not merely an economic activity but also a space where affective exchanges, tensions, and negotiations unfold. Integrated analysis of these dynamics is essential for informing policies on social protection, health promotion, and gender equity. Across the studies, domestic work—especially paid migrant domestic work—is consistently marked by social vulnerability, precarious employment conditions, physical strain, and mental health impacts, shaped by structural inequalities of gender, social class, and ethnicity.

Mental health and occupational stress emerge as the most robust and consistent findings across the reviewed literature. In contexts of extreme vulnerability, such as hospitalization settings, migrant domestic workers exhibited a high prevalence of brief psychotic episodes (66.7%) in Lebanon and Kuwait. Complementary analyses across geographic contexts reveal widespread symptoms such as depression, anxiety, worry, and loneliness. Stress levels were markedly elevated during periods of overseas employment and were strongly associated with working conditions and the quality of relationships with employers, creating a pattern of chronic emotional overload. These findings underscore that the historical precarization of reproductive labor extends beyond socioeconomic dimensions and manifests acutely in the psychopathology and psychological suffering experienced by these populations.

## 4 Discussion

Domestic work, both paid and unpaid, emerges in the reviewed studies as a central analytic category. It is performed predominantly by women across different age groups and family contexts and is shaped by dynamics of gender, social class, ethnicity, and nationality. It is important to underscore that class permeates all of these dynamics: the women portrayed in these studies are, in essence, poor women with marginalized life trajectories who seek opportunities beyond those available in their home contexts. The reality they encounter is paradoxical: despite the promise of improved living conditions, structural vulnerability remains constant, even in countries with vastly different socioeconomic realities. In other words, the conditions they face abroad resemble those they sought to escape. This discussion, therefore, centers on two major thematic findings of this review: (a) the context of domestic work within migration, and (b) its physical and psychological impacts, including related health disparities, which will be detailed below.

The studies demonstrate that domestic work, especially within migration contexts, constitutes a contemporary expression of structural inequalities across gender, class, ethnicity, and nationality. The literature shows that women's migration for domestic work is embedded in transnational flows driven by the pursuit of better living conditions but also shaped by systematic economic and political exclusion in their countries of origin (Bagley et al., 1997; Holroyd et al., 2001; van der Ham et al., 2015). These women migrate for both economic and emotional reasons, seeking to support their families, improve their children's educational opportunities, and distance themselves from poverty and violence. In doing so, they enter labor systems marked by asymmetry and inequality.

In contexts such as Hong Kong, Singapore, and Kuwait, contractual regimes for Foreign Domestic Workers are tied to residency rights. The central challenge is that access to employment does not translate into autonomy; the employer's home becomes an ambiguous space where living arrangements, labor demands, and subordination intersect (Lien Ha et al., 2018; Wickramage et al., 2017; Zahreddine et al., 2014). This configuration reproduces vulnerability, as job loss entails not only the loss of income but also the risk of deportation. Thus, migrant domestic work is often regulated by restrictive migration policies that deny workers basic rights such as freedom of movement, union protection, and access to public health services.

Ladegaard (2014) and Gorbán and Tizziani, 2018 show that the symbolic dimension of employer–employee relationships reinforces stereotypes of inferiority and dependence, legitimizing domination. Domesticity becomes not only structured by labor arrangements but also a site of meaning-making, shaping subjectivity through ambivalent experiences. At the same time, these spaces also generate unique forms of resistance and solidarity. Through community associations, migrant workers build networks of mutual support and find ways to reframe shared suffering, an example illustrated in the collective crying observed in group sessions among migrants in Hong Kong (Ladegaard, 2014).

Migration also entails identity reconfiguration, in which women oscillate between ties to their families of origin and integration into new cultural contexts. Amrith (2022) and Lien Ha et al. (2018) note that workers approaching retirement age in Singapore express emotional tensions linked to displacement and a lack of affective reciprocity. Many spend decades caring for other families, creating affective bonds in destination countries, though these relationships do not necessarily ensure protection or labor rights.

This ambiguity appears further in the comparative study by Amrith and Coe (2022), where caregivers working with older adults are described as “almost family.” Yet this label does not translate into security; high turnover remains the norm. The depiction of workers as family members exposes the utilitarian logic underlying affective ties in migrant domestic work, a dynamic that complicates workers' ability to raise concerns about the reality of their work.

Conversely, the study by Mkandawire-Valhmu et al. (2009), conducted in Malawi, illustrates how, even in extreme vulnerability, women demonstrate agency and symbolic resistance when confronting workplace violence. Through narratives of survival and solidarity, they affirm their dignity and identity. Thus, domestic work in migration contexts cannot be understood solely as an economic phenomenon; it is also a relational and affective space where meanings of belonging and power are disputed.

Taken together, the migration-related findings reveal that domestic work is structured by a logic of globalized inequality, sustained by the circulation of care work and the exploitation of the affective and bodily capacities of poor women from the Global South. Migrant domestic workers are simultaneously indispensable and invisible: central to the functioning of households and economies yet absent from narratives of citizenship and social protection. This issue cuts across the entire body of research analyzed and constitutes the backdrop for understanding the physical and mental health impacts that this work has on these women.

Alongside migration, the second analytic axis concerns the effects of domestic work, particularly in migrant contexts, on physical and psychological health, as well as the health disparities experienced by domestic workers. The body and the mind emerge as sites where structural inequalities and social suffering are inscribed. Across countries and time periods, the recurrence of physical symptoms, psychosocial distress, and somatic expressions of suffering indicate that domestic work is a high-cost occupation in terms of subjectivity and emotional burden.

Regarding physical health, studies consistently highlight the high prevalence of musculoskeletal pain, fatigue, and repetitive strain injuries associated with long working hours and the physical nature of domestic tasks (Cheung et al., 2016; Habib et al., 2011). The lack of rest, continuous exertion, and absence of adequate protective equipment lead to chronic pain and functional limitations, conditions often normalized by workers themselves as an inevitable part of “being a homemaker” or “a domestic worker.” This normalization reinforces the erasure of domestic work as labor and the subordinate status of those who perform it (Altschuler, 2004; Speiser et al., 2021).

The mental health findings are even more striking. Studies conducted in the Middle East and Southeast Asia describe acute psychological distress among migrant domestic workers, including depression, anxiety, brief psychotic episodes, and posttraumatic stress symptoms (Zahreddine et al., 2014; Wang et al., 2021). These manifestations are closely tied to experiences of isolation, restricted freedom, physical and sexual violence, and ruptured family ties. In many cases, distress is exacerbated by limited access to health care (Zahreddine et al., 2014).

Beyond explicit violence, subtle mechanisms of distress emerge from the emotional burden of caregiving. Research by Ho et al. (2018) and Heng et al. (2019) shows that caregivers of older adults, particularly migrants, often work more than 20 h per day, resulting in high levels of stress and physical/emotional exhaustion. The absence of boundaries between work and rest, intrinsic to domesticity, transforms the employer's home into a space of constant vigilance. These conditions align with sociological concepts of the colonization of subjectivity, where affection and obedience become job requirements (da Silva Moreira, 2025; Merlin, 2019; Viera, 2019).

The studies by Brian et al. (2019) and Hall et al. (2021a) conducted in Macau further show that mental health among migrant domestic workers is shaped by multiple social determinants. Feelings of discrimination and exclusion are strongly correlated with depressive and anxiety symptoms. Moreover, social capital, typically conceptualized as a protective factor, may paradoxically intensify distress when tied to pressures and expectations within domestic work (Brian et al., 2019). These findings point to the complexity of risk and protective factors, suggesting that, in the context of domestic work, the issue of illness takes on distinct contours and may also reproduce oppressive and stigmatizing norms.

Thus, the physical and psychological impacts of domestic work must be understood as outcomes emerging from relational contexts in which bodies, emotions, and social structures are deeply intertwined. The evidence converges on the understanding that workers' suffering is not an individual deviation but a socially produced phenomenon linked to precarious labor conditions, the invisibility of care work, and the marginalization of migrant women (Gorbán and Tizziani, 2018; Hall et al., 2021b; Wang et al., 2021). The literature suggests that effective interventions must integrate public policies addressing social protection, labor regulation, and culturally sensitive health promotion, recognizing the specificities of migrant domestic work and its implications for women's subjectivity.

In addition, prolonged exposure to chronic vulnerability factors (such as violence, subordination, and ruptured support networks) overloads neuroendocrine and immune systems, resulting in accelerated physiological wear (Moussiopoulou and Falkai, 2025). In this sense, acute psychological distress, including brief psychotic episodes, is not merely a psychological response but an extreme marker of biological harm induced by structural inequalities. The severity of these indicators highlights the need for future research to incorporate objective stress biomarkers to test the hypothesis of early biological aging and neurodegeneration in this population.

## 5 Final considerations

This scoping review reaffirms the critical importance of investigating the neurobiological and psychophysiological mechanisms underlying the relationship between precarious reproductive labor and illness among marginalized populations, particularly migrant and older women. The mapping of the literature revealed a high prevalence of acute psychological distress—including psychotic episodes in clinical settings and a substantial burden of anxiety and depression—which provides robust and consistent evidence of the impact of chronic vulnerability. These findings theoretically support the hypothesis of elevated allostatic load, whereby the chronic stress associated with precarization and violence manifests as physiological wear, thereby justifying the focus on internal biological mechanisms.

This study contributes by explicitly identifying the methodological gap in the field, which is rich in social and qualitative data but deficient in the measurement of biomarkers. The review advances the discussion by linking stigma and social precarization to severe clinical outcomes, offering a scientific basis for the development of public policies that recognize social oppression as a biological risk factor.

Future research should prioritize longitudinal studies integrating mixed methods, including the assessment of stress biomarkers and neuroimaging techniques, when feasible, to quantify the impact of allostatic load on brain function. Ultimately, scientific inquiry can no longer limit itself to describing suffering; it must also rely on incontrovertible neurobiological evidence to force the visibility of social oppression, transforming biological harm into an urgent demand for justice and political action in health.
