# Supplementary material for: Reproductive labor, social vulnerability, and female aging: a scoping review
Source: Front Sociol. 2026 Jul 20;11:1767534. doi: 10.3389/fsoc.2026.1767534 (PMC13430554; doi:10.3389/fsoc.2026.1767534)
Supplement: Supplementary file 1 [file Supplementary_File_1.pdf]

Supplementary Material 2

Table 2. Preferred Reporting Items for Systematic reviews and Meta-Analyses extension for Scoping Reviews (PRISMA-ScR) Checklist

SECTION	ITEM	PRISMA-ScR CHECKLIST ITEM	REPORTED IN METHODOLOGY / SECTION
**TITLE**	1	Identify the report as a scoping review.	Title
**ABSTRACT**	2	Provide a structured summary that includes...	Abstract
**INTRODUCTION**	3	Describe the rationale for the review...	Introduction (Rationale)
	4	Provide an explicit statement of the questions and objectives...	Introduction (Objectives)
**METHODS**	5	Indicate whether a review protocol exists...	Methods (Protocol and registration)
	6	Specify characteristics of the sources of evidence used as eligibility criteria...	Methods (Eligibility criteria)
	7	Describe all information sources in the search...	Methods (Information sources)
	8	Present the full electronic search strategy for at least 1 database...	Methods (Search strategy)
	9	State the process for selecting sources of evidence...	Methods (Selection of sources of evidence)
	10	Describe the methods of charting data...	Methods (Data charting process)
	11	List and define all variables for which data were sought...	Methods (Data items)
	12	If done, provide a rationale for conducting a critical appraisal...	Introduction / Methods (Eligibility criteria)
	13	Describe the methods of handling and summarizing the data...	Methods (Synthesis of results)

SECTION	ITEM	PRISMA-ScR CHECKLIST ITEM	REPORTED IN METHODOLOGY / SECTION
**RESULTS**	14	Give numbers of sources of evidence screened, assessed...	Results (Selection of sources of evidence / Figure 1)
	15	For each source of evidence, present characteristics...	Results (Characteristics of sources of evidence / Appendix Table 1)
	16	If done, present data on critical appraisal...	N/A
	17	For each included source of evidence, present the relevant data...	Results
	18	Summarize and/or present the charting results...	Results (Synthesis of results)
**DISCUSSION**	19	Summarize the main results...	Discussion (Summary of evidence)
	20	Discuss the limitations of the scoping review process.	Discussion (Limitations)
	21	Provide a general interpretation of the results...	Discussion (Conclusions)
**FUNDING**	22	Describe sources of funding...	Funding / Acknowledgments
