# Supplementary material for: Reproductive labor, social vulnerability, and female aging: a scoping review
Source: Front Sociol. 2026 Jul 20;11:1767534. doi: 10.3389/fsoc.2026.1767534 (PMC13430554; doi:10.3389/fsoc.2026.1767534)
Supplement: Supplementary file 2 [file Table_1.docx]

Supplementary Material

Table 1. Characteristics of the included studies

| **Study** | **Country** | **Population demographics** | **Study design** | **Key findings relevant to the objective of the study** |
| --- | --- | --- | --- | --- |
| Pennell and Smith (1959) | United States | Approximately 1,800 homemakers (home assistance workers) participated in the study. According to the data, these workers served about 2,200 families across 32 U.S. states and the District of Columbia. | A national survey conducted by the Public Health Service over one week in the first quarter of 1958. This was an observational, descriptive, and cross-sectional study aimed at mapping the characteristics and needs of families and the services provided. | Families were predominantly low-income; these services prevented institutionalization, reduced costs, and kept children and older adults at home. These groups include individuals with chronic illnesses or older adults, for whom community-based domestic services can save money by allowing them to remain at home instead of receiving long-term care in hospitals, nursing homes, or other institutions. |
| El-Hilu et al. (1990) | Kuwait | A total of 33 FDWs and 14 Lebanese women were included as a control group. The FDWs were of Ethiopian, Bangladeshi, Filipino, Tongan, Nepalese, and Sri Lankan origin. All were hospitalized for acute psychiatric episodes and assessed for sociodemographic, living, and mental health conditions. | A comparative study that administered the Brief Psychotic Rating Scale and the Clinical Global Impression at admission and discharge. Collected data included length of hospitalization, cumulative antipsychotic dosage, living conditions, history of abuse, and phenomenological observations, allowing comparisons between FDWs and the control group. | Findings from this study show that 66.7% of FDWs were diagnosed with a brief psychotic episode. There was a high prevalence of physical (37.5%), verbal (50%), and sexual abuse (12.5%). Striking clinical features included acute anorexia (39.4%), nudity (30.3%), and catatonia (21.2%). Despite symptoms similar to the control group, FDWs had shorter hospital stays and a tendency toward deportation. |
| Barling et al. (1993) | Canada | This study included 187 homemakers, mothers of children aged 2–3 years, who performed full-time domestic work or had fewer than 10 weekly hours of paid work outside the home. Mean age was 31.8 years, with 14.5 years of education and an annual income of US$44,478. Children: 34.5 months old, 48% girls and 52% boys. | Questionnaires assessing women’s experiences in the role of homemakers, including skill use, satisfaction, financial equity, and overload. Measures of maternal psychological well-being (positive/negative mood, cognitive difficulties) and parenting behavior (positive, punitive, rejecting) were also collected. | Maternal experiences within the domestic role indirectly affect children’s internalizing and externalizing behaviors, mediated by psychological well-being and parenting practices. The findings highlight that understanding how women experience the role of homemaker is essential, as it influences maternal health, parenting patterns, and children’s behavioral development. |
| Kemp et al. (1996) | United States | Sixteen women participated in this study, nine of whom were African American. Ages ranged from 23 to 55 years (five aged 23–24, six aged 28–40, and five aged 40–55). Participants resided in Orleans and Jefferson Parish, Louisiana, regions with a high proportion of female-headed households. | Semistructured interviews with key informants and focus groups with women enrolled in the JOBS welfare-to-work program, part of the 1988 Family Support Act and implemented in Louisiana in 1990. | Women receiving social assistance performed three kinds of labor: domestic work (for their own families), economic labor (paid jobs, including domestic service), and “eligibility work” (bureaucratic efforts required to obtain and maintain social benefits). The study challenges the stigma that beneficiaries “do not work.” |
| Bagley et al. (1997) | China | In this study, 600 Filipino domestic workers were interviewed. Most came from Luzon (74.8%), followed by Visayas (16.2%) and Mindanao (9%). Nearly half were single; among married participants, 206 had two or more children. Sixty percent had completed or partially completed college education, although many had never secured employment in the Philippines. | A cross-sectional study based on structured interviews to gather sociodemographic information, living conditions, work experience, mental health, and support networks. Cluster analysis was used to identify profiles associated with better or worse mental health among migrant domestic workers living in Hong Kong. | Two categories of women presented good mental health: single, educated women who migrated for adventure, and women with years of successful employment. Worse mental health was found among those who had experienced abuse and among women with debts and family problems. Despite stressors, the Filipino community built support networks and strategies for labor negotiation. |
| Holroyd et al. (2001) | China | This study included 290 Filipino domestic workers in Hong Kong, with a mean age of 32.1 years (SD = 7.4). Most had higher (51.1%) or secondary (42.4%) education; 47% were single and 41.5% married. Nearly half visited their families annually, and 43.7% had no children. | A simultaneous cross-sectional design. A validated English questionnaire, previously tested in a pilot study (n = 20), was administered to investigate health-related behaviors, locus of control, and social support. Information on living conditions, preventive habits, physical symptoms, and mental distress was collected. | The most common symptoms were initial insomnia, early morning awakening, worry, and loneliness. Persistent pain was frequent. Participants reported good dietary habits and low alcohol and nicotine consumption but weak adherence to preventive exams, such as Pap smears. Two-thirds attributed health behaviors to chance or to the influence of powerful people. |
| Mendelson (2003) | United States | Thirteen women of Mexican descent, moderately to highly acculturated, all mothers and over 18 years old, participated in the study. They were recruited through direct selection by the researcher, referrals from individuals familiar with the project, and recommendations from previously enrolled participants. All received formal explanations regarding their rights and confidentiality. | An ethnographic study using ethnographic interviews, participant observation, and field notes to explore cultural meanings of health-related domestic work. The goal was to understand cultural and contextual aspects of home-based health care among Mexican American women, identifying tensions and values embedded in their caregiving roles. | Two central categories emerged: motherhood and family caregiving. Participants reported overload and conflicts negotiating traditional cultural roles and contemporary demands, distinguishing between what they considered essential versus culturally unnecessary responsibilities. |
| Holroyd et al. (2003) | China | This study included 98 migrant domestic workers aged 24–45 years (M = 37.9, SD = 7.7), recruited from recreation centers. Most were married (82%), had children (91%), were non-smokers (88%), and had completed secondary or post-secondary education (66%). | A cross-sectional study employing snowball sampling due to challenges in accessing a random sample of migrant women. A structured questionnaire assessed cervical cancer knowledge and previous screening experiences, comparing responses between women with and without a history of Pap smears. | Women who had previously undergone Pap smears demonstrated greater knowledge of cervical cancer. The study recommends culturally adapted screening programs held outside standard business hours, along with providing health information in Tagalog, both written and oral, to increase access and adherence to preventive exams. |
| Altschuler (2004) | United States | Fifty-three ethnically and economically diverse women aged 55–84 from the greater Los Angeles area participated in this study. Most had completed high school or more, and nearly all were employed or seeking employment. Their life histories included structural inequalities such as racism and traumatic family experiences. | A qualitative study based on recorded in-person interviews. The study examined how older women perceive domestic work and unpaid responsibilities over the life course, considering the cultural, economic, and historical context shaping the meanings assigned to care and household tasks. | A shift occurred in the perception of domestic work, which lost importance with aging. The way care recipients reacted shaped the meaning of the activity. Experiences of racism and inequality reshaped how care was perceived, being seen both as a burden and as an opportunity for resistance and agency. |
| Mkandawire-Valhmu et al. (2009) | Malawi | Forty-eight young women (17–25 years, M = 21), former domestic workers seeking new employment in the field, participated in the study | Six focus groups using semistructured guides were conducted, followed by 10 individual in-depth interviews. A feminist approach prioritized participant safety and allowed spontaneous disclosures of violence and life experiences. | Their lives were marked by low educational attainment, early marriage, domestic violence, HIV risk, and workplace abuse, including beatings, food deprivation, and low wages. Despite this, they demonstrated resilience, breaking social norms and preserving dignity in the face of adversity. |
| van Wormer et al. (2011) | United States | Three older African American women who had worked in domestic service since childhood participated in the study, sharing their autobiographical memories. Their narratives reflect life trajectories marked by poverty, racism, and segregation, offering a personal perspective on the historical experiences of Black women in domestic occupations in the United States. | A qualitative study based on the analysis of personal narratives. Autobiographical accounts were examined through the lens of risk and resilience theories to understand coping strategies, resources mobilized to confront social and economic oppression, and the psychological and cultural impacts of racial segregation across the life span. | The narratives reveal not only survival amid racism and inequality but also the women’s ability to resist, rebuild meaning, and prevail. They show resilience processes that transformed painful experiences into strength and agency, highlighting learning, spirituality, and community solidarity as protective factors |
| Habib et al. (2011) | Lebanon | A total of 435 homemakers aged 18–62 participated in the study. All were previously or currently married and lived in Nabaa, a socioeconomically disadvantaged and ethnically diverse community on the outskirts of Beirut. | A cross-sectional study (2006) using a structured questionnaire. The sample was derived from earlier studies (2002–2003), ensuring continuity and comparability. | Study findings indicate that 77% reported musculoskeletal pain in the previous year. Risk factors included household stress, number of children, fatigue, long working hours, improper postures, and repetitive movements. Physical and psychosocial conditions interacted, revealing a high prevalence of pain associated with domestic work. |
| Ladegaard (2014) | China | The study analyzed life histories of 230 FDWs from Indonesia and the Philippines living in Hong Kong. Participants were recruited at the Bethune House shelter, where they sought support after experiences of abuse or fleeing employers, and took part voluntarily in sharing sessions. | A qualitative study with ethnographic inspiration, based on 55 sharing sessions (60–90 minutes), 35 of which were transcribed and translated when needed. The analysis identified 89 crying events and conducted discourse analysis on six selected excerpts to understand the social and psychological functions of collective crying. | Crying was interpreted as discursive performance and catharsis, challenging the idea that it is merely an expected emotional expression. Analyses show that crying promotes group cohesion, validates suffering, and mobilizes support. The study proposes foundations for a psychosocial theory of crying in migrant discourse. |
| Gorbán and Tizziani (2014) | Argentina | Twenty domestic workers in Buenos Aires were interviewed in depth in 2009. Participants were recruited through unions and associations. Additional observations were conducted at two city locations over four months. | A qualitative study based on in-depth interviews and participant and informal observations in organizations associated with domestic work. The objective was to examine implicit social hierarchies in employer–worker relations. | Employers construct stereotypes of domestic workers’ social inferiority, legitimizing their dominant position in the labor relationship. The study also reveals tensions and ambiguities in this process, showing subtle mechanisms of reproducing inequality. |
| van der Ham et al. (2014) | China | This study included 500 Filipino women (18–60 years old) who had completed at least one overseas domestic work contract. The sample was distributed across four locations (La Union, NCR, Davao, Cebu) representing the country’s three main regions. Purposive sampling was complemented by snowball sampling. | An exploratory study with both quantitative and qualitative components. Questionnaires assessed stress and coping across different migration stages. | The study found that women experienced greater stress while working abroad. In the Philippines, coping was related to financial issues. In the destination country, stress was linked to loneliness, work conditions, and employer relations. The study highlights phase-specific and transnational coping dynamics. |
| Zahreddine et al. (2014) | Lebanon | This study included 33 FDWs — Ethiopian, Bangladeshi, Filipino, Tongan, Nepalese, and Sri Lankan — and 14 Lebanese women as a control group. All were hospitalized for acute psychiatric treatment and assessed for sociodemographic data, living conditions, and clinical profiles. | A comparative study that used the Brief Psychotic Rating Scale and Clinical Global Impression at admission and discharge. Analyses included cumulative antipsychotic dosages, hospitalization length, and histories of physical, sexual, and verbal abuse, as well as phenomenological observations specific to migrant domestic workers receiving inpatient treatment. | In this study, data indicate that 66.7% of FDWs were diagnosed with a brief psychotic episode. High rates of abuse were identified, along with striking phenomena such as acute anorexia (39.4%), nudity (30.3%), catatonia (21.2%), and pregnancy delusions (12.1%). Despite severity similar to the control group, FDWs had shorter hospitalizations and a tendency toward rapid deportation. |
| van der Ham et al. (2015) | China | The study involved migrant Filipino domestic workers. A total of 500 women completed questionnaires and participated in focus groups, representing diverse migration trajectories and experiences in overseas domestic work. | An exploratory mixed-methods design combining quantitative questionnaires on stress, well-being, and personal resources with qualitative procedures. Data were further deepened through a workshop and two focus groups, which helped validate and expand the initial findings. | Participants reported relatively good well-being despite high stress. Social coping strategies and spirituality were central resources. Social networks and employers strongly influenced the resources available. Social resources were more strongly associated with stress and well-being than personal resources. |
| Bernadas and Jiang (2016) | China | Twenty Filipino domestic workers in Hong Kong participated in focus groups designed to investigate patterns of health information seeking. Participants were temporary or permanent migrants working in the global domestic work market, exposed to vulnerabilities and health inequities related to migration processes. | A qualitative study using focus group discussions to examine how women access health information and which social actors influence their preventive behaviors. The analysis aimed to understand the role of social networks, employers, and religious practices in shaping health attitudes and practices among migrants. | Employers emerged as relevant health actors, online social networks functioned as spaces for participatory health promotion, and religious beliefs encouraged healthy behaviors. The study highlights the potential of these networks and practices for more inclusive and culturally sensitive health interventions. |
| Cheung et al. (2016) | Australia | Fifteen homemakers with upper-limb RSI participated in the study, with a mean age of 58.5 years. Most were married, lived with their children, and performed most household tasks. | A constructivist grounded theory approach was used. Data were collected through in-depth interviews and analyzed using line-by-line, focused, and axial coding with constant comparison. | Decision-making around domestic tasks involved emotional attachment, cognitive decisions, and emotional influence. Women with RSI faced significant emotional barriers to changing their practices, even when recognizing the need to do so. |
| Wickramage et al. (2017) | Sri Lanka | Twenty migrant Sri Lankan women who had worked as domestic workers in the Middle East and returned to the country participated in the study. All were referred to the Medico-Legal Department of the National General Hospital between October 1, 2014 and February 31, 2015. | A cross-sectional study conducted with Sri Lankan migrant domestic workers who had returned from the Middle East and were assessed through medicolegal examinations. The objective was to identify manifestations of abuse and provide an exploratory overview of the forms of violence faced by this population. | Abuse against migrant domestic workers manifests in multiple forms: physical, financial, verbal, emotional violence, and neglect. Despite limitations regarding generalizability, the findings point to the need for future research with larger populations and affirm the commitment of public policy structures to preventing forms of violence targeting this population. |
| Ferrer (2017) | Canada | Six older Filipino domestic workers who had immigrated participated in the study, discussing their experiences of migration, work, retirement, and aging. | A qualitative study based on six in-depth interviews. The focus was to understand participants’ life trajectories and challenges. | Participants faced poverty as they approached retirement, and state pensions were insufficient. As a survival strategy, many needed informal work, and caregiving duties were passed down across generations. |
| Ho et al. (2018) | China | Eleven women aged 27–57, with 2–15 years of experience caring for older adults, were voluntarily recruited from local NGOs and personal networks. Seven were from the Philippines and four from Indonesia. | A qualitative study drawing on Heideggerian hermeneutic phenomenology and van Manen’s methods to investigate lived and socially conditioned experiences of women caring for older adults, considering the notion of “being-in-the-world” and the transformation of reality through cyclical interpretation. | The study identified two central themes: the commodification of foreign domestic workers as functional caregivers and the reciprocity of companionship. Findings reveal a transition from task-oriented relations to companionship-based ties, with performative and affective dimensions grounded in moral values. |
| Lien Ha et al. (2018) | Singapore | Fifty migrant women of various nationalities (primarily from the Philippines, as well as Indonesia, India, and Sri Lanka), aged 48–68, living and working in Singapore for 18–35 years, participated in the study. The research focused on the older generation of domestic workers nearing retirement. | A qualitative ethnographic study conducted since 2018, using in-depth interviews and a life-course approach. The research analyzed age transitions relationally and contextually, exploring subjective experiences in relation to age norms imposed by employers and the state in Singapore. | Workers planned, anticipated, or avoided discussing aging and retirement, affected by the mandatory return at age 60 due to economic vulnerability. Mixed emotions emerged as they reassessed caregiving, relationships with employers, and ties with families and communities. The research highlights tensions between citizenship, gender, care, and temporal boundaries over the life course. |
| Álvarez-López (2019) | Chile | Three older domestic workers participated in the study. | An ethnographic study exploring women’s cleaning practices to understand how they position themselves in terms of class and gender and the meanings attributed to these tasks. | Women expressed their subjectivity and constructed working-class womanhood through their cleaning practices. Negotiation capacity over cleaning standards was lower when the outcomes of their labor were publicly visible. |
| Heng et al. (2019) | Singapore | Eleven foreign domestic workers in Singapore, with a mean age of 35 years, participated. They were employed to care for adults over 60 years who needed assistance with daily activities. | A qualitative study using purposive sampling. In-depth, in-person interviews were conducted until data saturation was reached. Interviews were transcribed and analyzed using thematic analysis. | Participants spent about 20 hours a day providing care and faced multiple challenges. The main themes were: “Balancing Caregiving and Additional Responsibilities,” “Facing Challenges,” “Coping Strategies,” and “Seeking Support.” Workers felt overloaded, without rest, and stressed, especially when caring for older adults with behavioral issues. |
| Brian et al. (2019) | China | A total of 131 migrant Filipino domestic workers in Macao participated, with a mean age of 39.7 years (SD = 8.3; range 21–59). The sample was recruited through snowball sampling. | A cross-sectional study (March–October 2016). Self-reported data collected via tablets included PHQ-9 scores for depressive symptoms and measures of anxiety, discrimination, and social capital. Correlations and multivariate regressions were conducted. | Research findings showed that the “discrimination” factor correlated positively with depression (*r*_s_ = .43) and anxiety (*r*_s_ = .42). Cognitive social capital was negatively associated with symptoms but paradoxically intensified the impact of discrimination on depression and anxiety. Results indicate that limited social resources may, in some contexts, exacerbate psychological distress. |
| Wong et al. (2020) | Singapore | Forty Filipino women aged 23 or older, English-literate, with at least nine years of education, and able to participate in a four-week course in Singapore were included. They were recruited through social networks and the NGO HOME and met eligibility criteria for in-person training. | A randomized experimental study with an intervention group (CBT training for paraprofessionals) and a waitlist control group. Outcomes included depression literacy, CBT knowledge, stigma, and attitudes toward seeking professional help, assessed before, after, and two months post-training. | No significant differences were observed between groups, but both demonstrated significant improvement in depression literacy, CBT knowledge, and help-seeking attitudes. Improvements were maintained at the 2-month follow-up. Participants reported high satisfaction. Findings suggest the need for program adjustments to increase effectiveness. |
| Speiser et al. (2021) | United States | Focus groups included nine women (mean age = 48.78, SD = 6.72) from South America (n = 5), Mexico (n = 1), El Salvador (n = 1), and the Dominican Republic (n = 2). Mean residence in the United States was 18.78 years. More than half had over 10 years of cleaning experience. | A mixed-methods study conducted in two phases: (1) three focus groups (N = 15) exploring cleaning-related knowledge, attitudes, and practices based on training experiences; and (2) a cross-sectional 43-item questionnaire assessing quantitative aspects of product and equipment use. | Latina women working in the cleaning sector face significant social and occupational barriers, such as lack of safety training, inconsistent use of protective equipment, and low literacy levels. Findings highlight the need for targeted interventions that provide training and adequate resources to support this essential workforce. |
| Wang et al. (2021) | China | A total of 1,375 Filipino domestic workers over 18 years old, holding valid work visas and recruited via targeted sampling in Macao (SAR), participated in the study, representing approximately 10% of the local Filipino population. Participants provided informed consent and received monetary compensation. | A cross-sectional study with self-administered tablet questionnaires (November 2016 to August 2017). It investigated the comorbidity between PTSD symptoms and sleep dysfunction, identifying bridge symptoms linking different symptom clusters. | Symptoms with the strongest bridging effects included concentration difficulties, impulsivity, irritability, and sleep disturbances. Connections between symptom clusters showed that sleep dysfunction is strongly linked to PTSD. Findings suggest specific intervention targets for comorbidities among trauma-exposed migrants. |
| Hall et al. (2021a) | China | This study included 1,388 Filipino domestic workers over 18 years old, holding work visas or residence cards in Macao (SAR), recruited through targeted sampling between November 2016 and August 2017. Data on demographic characteristics, health behaviors, and anthropometric measures were collected. | A cross-sectional study using tablet-based self-report, objective measurements of height and weight, and geocoding of residences and restaurants via ArcGIS. Multivariate analyses examined associations between fast-food restaurant density and participants’ weight status (overweight/obesity). | The study identified that about 64% of participants were overweight or obese. Greater density of fast-food restaurants within a 0.5-mile radius of the residence significantly increased the odds of overweight/obesity (aOR = 1.07). Results indicate the need for healthier work environments with adequate access to nutritious food. |
| Hall et al. (2021b) | China | The study included 1,375 Filipino and 369 Indonesian participants, all over 18 and legally employed as domestic workers in Macao (SAR). All provided informed consent. After excluding incomplete responses, data from the 1,375 Filipino and 367 Indonesian participants remaining were analyzed; recruitment was conducted through targeted sampling. | A community-based study analyzing PHQ-9 scores to assess depressive symptoms. CFA and MGCFA were conducted to test measurement invariance across samples. Five structural models were compared to identify the best representation of depression factors. | The two-factor model (cognitive/affective and somatic) best fit both samples. Somatic symptoms were prominent, especially loss of energy. Suicidal ideation showed low means. The model shows that depression among Filipino and Indonesian domestic workers may be differentiated between cognitive/affective and somatic dimensions. |
| Amrith (2022) | Singapore | This study included 50 migrant women from various nationalities (primarily from the Philippines, also from Indonesia, India, and Sri Lanka), aged 48–68, living and working in Singapore for 18–35 years. The research focused on the older generation of domestic workers nearing retirement. | A qualitative ethnographic study conducted since 2018, using in-depth interviews and a life-course approach to examine relational and contextual age transitions and subjective experiences relative to state and employer-imposed age norms in Singapore. | Workers planned, anticipated, or avoided discussing aging and retirement, affected by the mandatory return at age 60 due to economic vulnerability. Mixed emotions emerged as they reassessed caregiving, relationships with employers, and ties with families and communities. The research highlights tensions between citizenship, gender, care, and temporal boundaries over the life course. |
| Amrith and Coe (2022) | United States | The study included 59 African caregivers in the United States, 25 patient families, and staff from 15 home-care agencies. The second author, Megha, accompanied migrant domestic workers from South and Southeast Asia in Singapore, the Philippines, and Sri Lanka. | A qualitative and comparative study based on collaborative ethnography. The research connected both contexts to explore care relations, migration, and kinship. | The research suggests that care workers are viewed as “flexible kin” during employment but ultimately treated as “disposable kin” in an essentialist sense of kinship. This fluctuation denies them obligations and post-employment support, such as inheritance or eldercare, allowing employers and the state to avoid responsibilities. |
| Xhaho et al. (2025) | Greece | Nineteen Albanian migrant women aged 22–59 employed as domestic workers in Greece participated in the study. Most were married, had children, and had arrived through family reunification, later becoming irregular migrants. Many had worked for the same families for over 15 years. | A qualitative study based on 19 biographical interviews conducted in Albanian in Greece between March 2014 and November 2016. The study adopted a micro-level approach to understanding in detail the experiences of Albanian migrant domestic workers. | Social and economic relations were shaped by the emotional and intimate dimension of domestic work. Affective bonds created home-like environments but also ambiguities: while some women felt welcomed, others saw such ties as burdens that hindered rights claims and reinforced vulnerability. |

**Abbreviations:** aOR = adjusted odds ratio; CBT = cognitive-behavioral therapy; CFA = confirmatory factor analysis; FDW = foreign domestic worker; MGCFA = multigroup confirmatory factor analysis; NGOs = nongovernmental organizations; PHQ-9 = Patient Health Questionnaire-9; PTSD = post-traumatic stress disorder; RSI = repetitive strain injury; SD = standard deviation.
